# Analytic-agent cyber dynamical systems analysis and design method for modeling spatio-temporal factors of malware propagation in wireless sensor networks

**DOI:** 10.1016/j.mex.2018.10.005

**Published:** 2018-10-16

**Authors:** ChukwuNonso Nwokoye, Ikechukwu Umeh

**Affiliations:** Department of Computer Science, Nnamdi Azikiwe University, Nigeria

**Keywords:** Agent Oriented Software Engineering/Agent Oriented Analysis and Design Method, Analytical benchmark model, Agent model, Equilibrium generation, Continuous validation, High level conceptual model

## Abstract

Modelers often apply analytical (differential equation-based) epidemic models that mostly characterize the behavior of the network compartments with passage of time. Beyond temporal characterization, agent modeling promises the achievement of relevant spatial (stochastic and heterogeneous) representations. Arising from the combination of the prevalent analytical and agent methods (gleaned from extant literature) is a new method called the *Analytic-Agent Cyber Dynamical Systems Analysis and Design Method (A^2^CDSADM);* a modification of the Agent Oriented Analysis and Design (AOAD). Using hypothetical wireless sensor network (WSN) cases, A^2^CDSADM alleviates the lack of field data/lack of real geographical locations of the occurrence of particular cases by creating an *analytical benchmark model* for initial validation of the resulting agent model and ensures its easy modifiability and reproducibility. More so, it helps achieve the complementary/generative contribution of agent modeling, diminishes the less-tractable nature of representing/analyzing WSN spatial features and provides a formalized method for performing comparative epidemic studies. Also, A^2^CDSADM covers the additional features for:

• Generating the (analytical) equilibriums of WSN.
• Performing continuous validation (at several points) in order to ensure model accuracy/suitability for real-world decision making.
• Creating a high level conceptual model containing the envisaged WSN features to be represented.

## Graphical abstract

**Figure:**
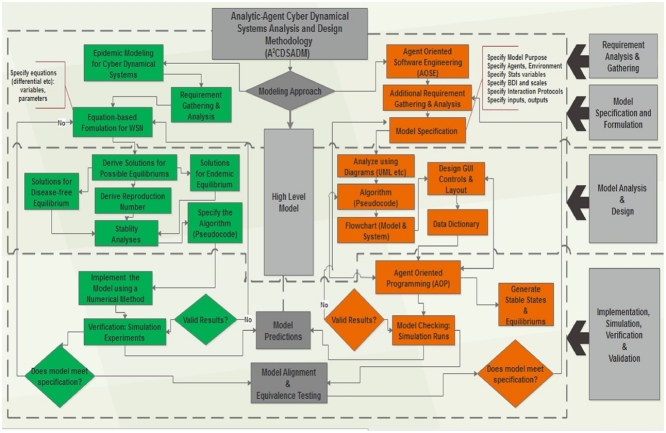

Specifications Table

**Table:** 

Subject area	*Computer Science*
More specific subject area	*Agent Simulation and Modeling*
Method name	*Agent Oriented Software Engineering/* *Agent Oriented Analysis and Design Method*
M. Wooldridge, P. Ciancarini, Agent-oriented software engineering: The state of the art, in: Agent-oriented software engineering. Springer Berlin Heidelberg, 1–28, 2001	*Summarily, they asserted that the major roles played by formal methods in software engineering include; the specification of systems; for directly programming systems; and in the verification of systems. This implies that specification, programming and model verification are the stages of their methodology.*

## Method details

In computational modeling, most researchers represent and illustrate dynamical systems such as networks in equilibrium (or steady state) or going between steady states. However, agent modeling (a form of individual based models (IBMs)) can complementarily add to the equation-based approaches. Where equation-based approaches allow researchers to characterize the steady states of a dynamical system, multiagent modeling permits the strong likelihood and practicability of producing those steady states. Perhaps the productive and supplementary aspect constitutes the major advantage of complementing analytical modeling with agent-based modeling. This is because agent modeling employs simple rules that result in diverse complex behaviors of a real world phenomenon. Since emergence and complexity are its essential concepts, agent modeling goes beyond the steady state-orientation of analytical modeling to the investigation of a system’s robustness and adaptability. However, there is no formalized method for achieving the productive and supplementary contribution of agent-based modeling to analytical modeling methods.

**Fig. 1:**
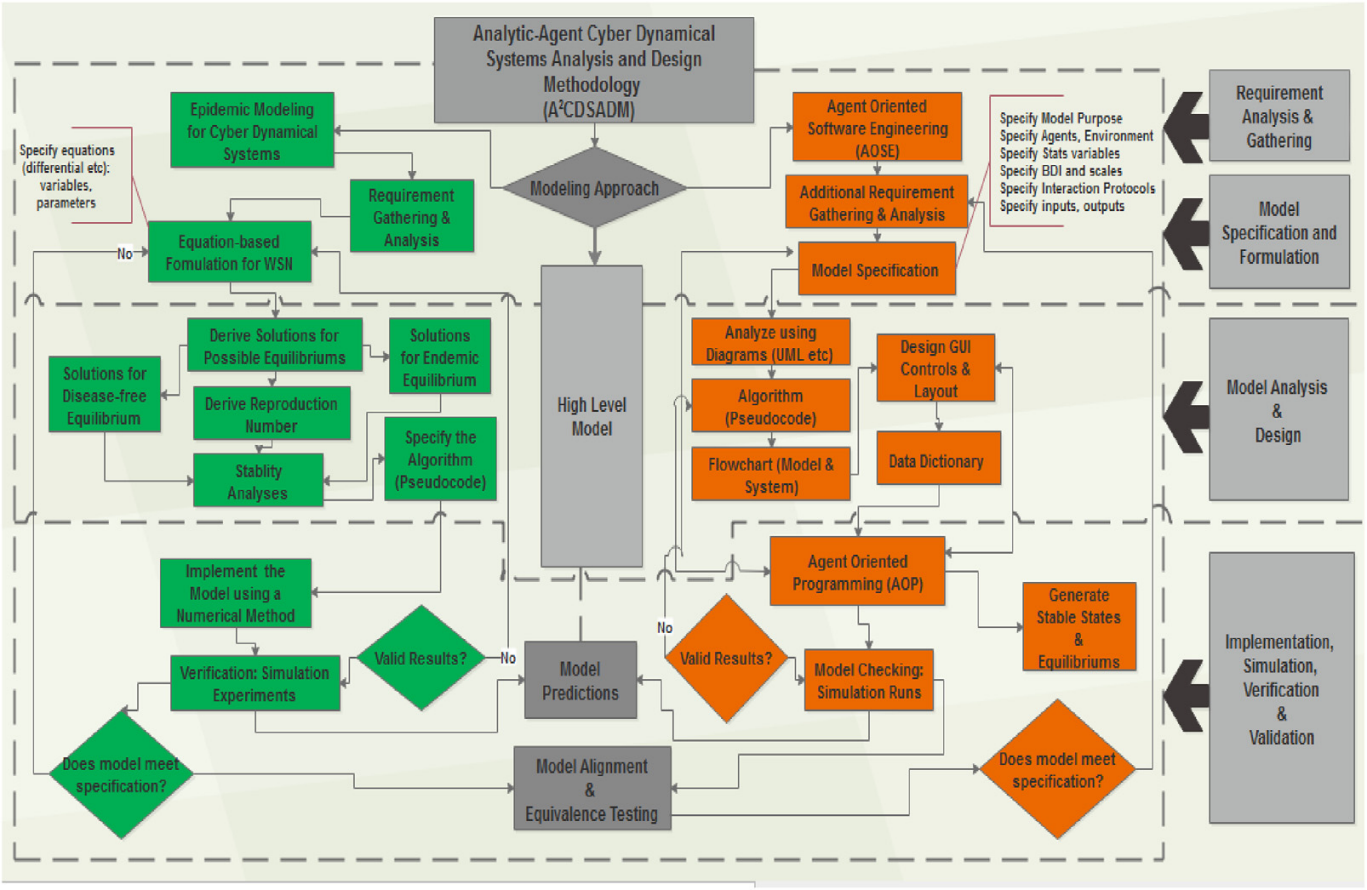
Diagrammatic representation of the A^2^CDSADM.

For the equation-based (analytical) approach we adopt a method gleaned from the extant literature on network epidemiology (which we referred to as) the *Epidemic Modeling and Analysis of Cyber Dynamical Systems*. For the Agent-based approach we employ the *Agent Oriented Software Engineering* approach [1] which applies the Agent Oriented Programming (AOP) in its implementation. Merging the two approaches is very necessary in order to employ their respective strengths. This merger resulted to a new method, referred to as the ***Analytic-Agent Cyber Dynamical Systems Analysis and Design Method (A2CDSADM)*** (Fig. 1). While analytical modeling achieve the development of traditional analytical WSN models by compartmentalization of nodes according to their health status, agent modeling would enable the building of agent simulators with more spatial capabilities using simple rules. The developed simulator would go beyond representing some characteristics of our proposed models to easily characterizing factors such as mobility, packet transmission, multi-group malicious code transmission, carrying capacity, sensor monitoring, sensor (daily) ageing, life span and death etc.

Essentially, the method complements the creation of accurate agent based models with a benchmark analytical model (BAM) involving a system of ordinary differential equations. This BAM aids foundational validation of the first agent model before subsequent modification. Note that the creation of a BAM became necessary because it was found that in some studies that attempt to validate agent models of malware propagation with an analytical model, the obsolete SIR model [2] was used; such works include [[3], [4], [5], [6], [7], [8], [9], [10]]. One of the reasons for doing so is the well known nature of the SIR model; another reason might be due to the unavailability of an equivalent analytical model. This is not only inadequate but inappropriate because the SIR model does not involve other complexities that may exist in a real world epidemic scenario. In our study for example, the SIR model does not include the dynamical behaviour of the exposed and the vaccinated compartment as seen in the SEIR-V model.

The well known nature of the SIR model is based on simplicity, clarity, ease of implementation [4] and proven accuracy. However, these advantages could be achieved using the proposed method. Specifically, an analytical model of interest could be modified and validated before it is used to validate the resulting agent based model. On the question of validation, note that the essentiality of **A^2^CDSADM** becomes clearer and more vivid in cases where there is the lack of real world (field) data or the lack of real geographical locations of the occurrence of particular cases. The analyst/modeler may resort to some sort of cross-validation; to develop a richer model alongside a general model i.e. creating a *benchmark* with the traditional analytical model which will be used for comparing and validating the agent model. This type of validation (in **A^2^CDSADM)** is not only helpful, but verily expedient if; 1. there exists no analytical model that closely mimics the proposed agent model and 2. part of the modeler’s objectives is the incremental modification of the agent model (simulator) to mimic real world WSN scenarios (the additional information section addressed validation processes and its attendant issues).

The new method combines the strength/benefits as well as the essential aspects of the two methods. Essential aspects imply those steps that allow the achievement of the complimentary and generative role of agent-based method to the traditional analytical approach as well as other software engineering approaches that will enhance modification and reproducibility of resulting models.

We chose the differential equation modeling method as the analytical approach for our new method because they are popular and they dominate the literature on malware propagation. To buttress this viewpoint, Martín [11] posits that, “… most of the mathematical models designed to study the propagation of malware are based on the use of differential equations”. Our decision to use a differential equation modeling method (gleaned from literature) was founded on this rationale. On the other hand, we choose agent modeling because it offers more benefits than the cellular automata approach – another form of individual based models. Individual-based models are briefly reviewed at the last section of the paper.

From the graphical abstract, the methodology is vertically divided into; 1. Requirement Gathering and Analysis; 2. Model Specification and Formulation; 3. Model Analyses and Design and 4. Implementation: Simulation, Verification and Validation. To use the method one can perform the analytical modeling first and then perform the agent modeling before generating predictions and model alignment. On the hand one can perform all the activities that constitute each vertical division (for both modeling approaches), then finally perform model predictions and model alignment.

To explain the steps of the new method, two hypothetical cases studies are used for analytical and agent modeling. On analytical modeling, a hypothetical case study of a wireless sensor network (WSN) model adapted from [12] and modified to include density and transmission range [13], is used. Then for agent modeling, we will use a case of representing the essence of the model in [12,13], with even more WSN features.

In case the analyst wants to perform a comparative study i.e. modifying an older model in order to cater for overlooked/ignored but relevant factor of a network, the instances of similarities/differences (and its effects) should be clearly specified. Therefore, in the light of all the benefits and differences, one could say that our A^2^CDSADM is a better expression of the Agent Oriented Analysis and Design method/Agent Oriented Software Engineering.

## Analytical (equation-based) modeling

Under the equation-based approach we employ a popular method gleaned from the literature on studying malicious code propagation in networks [12,[14], [15], [16], [17], [18]]. This method can be referred to as the epidemic modeling and analysis of dynamical systems. Using this method, analysts treat the networked system like a dynamical system that possesses equilibrium points. The equilibrium points are later investigated. The steps/stages of this method are discussed below:

### Initial requirement gathering and model formulation

Havey [19] posits that the first step in the development of a model is to study and be thoroughly familiar with the operating realities of the system to be modeled, if the system is available or the system whose operation is nearest to it, if the system is not available. In the light of this assertion, we studied relevant details of threat (worm, virus and trojan) as well as available literature on threat/infection propagation and containment in networks. Equipped with the required information, the real system is then reduced firstly to a schematic representation (continuing equation), and thereafter to a system of differential equation. Note that the differential equations are used here to depict the rate of change of WSN parameters with respect to time.

In Nwokoye et al. [13], we represent worm attack in wireless sensor network using the Susceptible–Exposed–Infectious–Recovered–Susceptible with a Vaccination class (SEIRS-V); its parameters are presented in Table 1. The total population *N (t)* represents the nodes in the Wireless Sensor Network which is subdivided into Susceptible, Exposed (latent), Infectious (contagious), Recovered (temporarily immune), Vaccinated (immunized) denoted by S(t), E(t), I(t), R(t) and V(t). This implies that S (t) + E (t) + I (t) + R (t) + V (t) = N (t).

**Table 1:** Parameter Description for SEIR–V Model.

Parameters	Name	Meaning
*σ*	sigma	Distribution density
$r_{0}^{2}$	r	Transmission range
$\sigma \pi r_{0}^{2}$	–	Effective contact with an infected node for transfer of infection
λ	lambda	Inclusion rate of nodes into the sensor network population
β	beta	Infection contact rate
τ	tau	Death rate of nodes due to hardware or software failure
ω	omega	Crashing rate due to attack of malicious objects (in this case worm)
θ	theta	Rate at which exposed nodes become infectious
ν	nu	Rate of recovery
φ	phi	Rate at which recovered nodes become susceptible to infection
ρ	rho	Rate of vaccination for susceptible sensor nodes
ξ	xi	Rate of transmission from the Vaccinated compartment to the Susceptible compartment

**Fig. 2:**
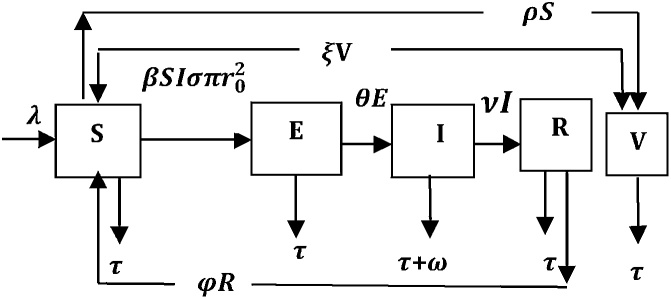
Schematic diagram for the flow of worms in sensor networks [13].

The transition rules governing model dynamics are presented below. Using these rules we generated the schematic diagram of transitions (Fig. 2) as well as the resulting system of equations.

Step 1: Node Deployment/Inclusion and Network Initialization – The sensor nodes are uniformly randomly deployed (at the inclusion rate of λ) with a node density of σ over a sensor field and since they are equipped with antennas, information collection/communication between sensors is done over a maximum transmission range $r_{0}$.

Step 2: State Initialization for Sensor Nodes – The newly deployed sensor nodes are in the Susceptible (S) state and are removed as a result of hardware/software failure at the rate of τ.

• Case 1: If there is worm attack and an effective spread of the worm infection on the order of $\sigma \pi r_{0}$, sensor nodes changes their states from Susceptible to Exposed state (where sensors are not fully infectious) at the combined rates of $\beta SI\sigma \pi r_{0}$. Otherwise the sensor network population approaches the carrying capacity denoted by λ/τ. Nodes are removed as a result of hardware/software failure at rate τ.
• Case 2: If latency time has elapsed, sensor nodes in the Exposed (E) state changes to Infectious (I) state (where sensor nodes become fully infections) with a transition rate of θ; nodes are probably removed from the network as a result of hardware/software failure at τ and worm infection at rate ω.
• Case 3: If the sensor network is equipped with functional and improved antivirus capability, sensor nodes in the Infectious (S) state change to the Recovered (R) state at a transition rate of ν, where some nodes are probably removed as a result of hardware/software failure at the rate of τ and Recovered (R) nodes revert back to the susceptible (S) state after a period of immunity (1/φ) at transition rate φ.
• Case 4: If sensor network possess a functional immunization capability, sensor nodes changes from Susceptible (S) state to Vaccinated (V) state at transition rate ρ, where some nodes are removed as a result of hardware/software failure at the rate of τ and Vaccinated (V) nodes reverts back to the susceptible (S) state at transition rate ξ after a period of immunity (1/ξ).

The modified SEIRS-V model is represented using the following system of differential equations;

(1) $$\;\begin{array}{c} \dot{S}=\lambda -\;\beta SI\sigma \pi r_{0}^{2}-\tau S-\rho S+\varphi R+\xi V\\ \dot{E}=\beta SI\sigma \pi r_{0}^{2}-\;\left(\tau +\theta \right)E\\ \begin{array}{c} \dot{I}=\;\theta E-\left(\tau +\omega +\nu \right)I\\ \dot{R}=\nu I-\left(\tau +\varphi \right)R\\ \dot{V}=\rho S-\left(\tau +\xi \right)V \end{array} \end{array}$$

Accurate description of a real world phenomena might motivate a comparative epidemic studies (like in the case study), wherein an older model is modified. The analyst should establish the parameters/variable that needs to be either added/removed in order to initiate the comparative study. Specifically, the authors of [13] noted that the effective contact rate in a real world WSN can be beyond βSI (as presented in [12]), if the density and transmission range is considered. This observation motivated the inclusion of ${{\sigma \pi r}_{0}}^{2}$ in the schematic diagram for the flow of worms in sensor networks and in the formulated model (system of differential equations). Note that before now it has been established [[20], [21], [22], [23], [24]] that in modeling epidemics wireless sensor networks there exists a certain range (*r*) and distribution density (σ) (described in Table 1). As Tang & Mark [20] puts it, “since the nodes are uniformly randomly distributed with density *σ*, each infected node can contact on the order of ${\sigma \pi r}_{0}^{2}$ neighbor nodes”. Additionally, βwhich is the infection capacity/contact rate represents the probabilistic rate of getting infected in a contact between an infective (*I*) and a susceptible (*S*) node. Therefore, β depends on the worm’s infectivity and communication range of the existent protocol. The implication is that the effective contact rate for worm transmission is $\beta SI{\sigma \pi r}_{0}^{2}$.

### Finding the equilibrium states

In studying disease spread it has been an established that there exists two equilibriums namely; disease free equilibrium and endemic equilibrium [12,[15], [16], [17],^25^,^26^]. Disease free equilibrium is also called the infection-free equilibrium and it describes the absence of infection/disease/threat in the network; while endemic equilibrium describes the presence of infection/disease/threat in the network. In the hypothetical case study, we derived the following solution for the equilibrium points;

The solutions of the Worm-free equilibrium $E_{0}^{F}=\;(S_{0}^{*},E_{0}^{*},\;I_{0}^{*},\;R_{0}^{*},{\;V}_{0}^{*})$ i.e.

(2) $$S_{0}^{*}=\frac{\lambda \left(\xi +\tau \right)}{\tau \left(\xi +\rho +\tau \right)};E_{0}^{*}=0;\;I_{0}^{*}=0;R_{0}^{*}=0;\;V_{0}^{*}=\frac{\lambda \rho }{\tau \left(\xi +\rho +\tau \right)}\;$$

The solutions of the Endemic equilibrium $E_{1}^{E}$ = ($S_{1}^{*}$,$\;E_{1}^{*}$,$I_{1}^{*}$, $R_{1}^{*}$, $V_{1}^{*}$) for the circular strip topology i.e.

(3) $$\begin{array}{c} \begin{array}{c} S_{1}^{*}=\frac{\left(\theta +\tau )(\nu +\tau +\omega \right)}{\beta \theta {\sigma \pi r}_{0}^{2}}\\ E_{1}^{*}=\frac{\left(\tau +\varphi )(\nu +\tau +\omega )(\lambda -\frac{\tau (\theta +\tau )(\xi +\rho +\tau )(\nu +\tau +\omega )}{\beta \theta (\xi +\tau ){\sigma \pi r}_{0}^{2}}\right)}{\theta \tau (\nu +\tau +\varphi )+\theta (\tau +\varphi )\omega +\tau (\tau +\varphi )(\nu +\tau +\omega )}\\ I_{1}^{*}=\frac{\left(\tau +\varphi )(\theta \lambda -\frac{\tau (\theta +\tau )(\xi +\rho +\tau )(\nu +\tau +\omega )}{\beta (\xi +\tau ){\sigma \pi r}_{0}^{2}}\right)}{\theta \tau (\nu +\tau +\varphi )+\theta (\tau +\varphi )\omega +\tau (\tau +\varphi )(\nu +\tau +\omega )} \end{array}\\ R_{1}^{*}=\frac{\nu (\theta \lambda -\frac{\tau (\theta +\tau )(\xi +\rho +\tau )(\nu +\tau +\omega )}{\beta (\xi +\tau ){\sigma \pi r}_{0}^{2}})}{\theta \tau (\nu +\tau +\varphi )+\theta (\tau +\varphi )\omega +\tau (\tau +\varphi )(\nu +\tau +\omega )}\\ V_{1}^{*}=\frac{\rho (\theta +\tau )(\nu +\tau +\omega )}{\beta \theta (\xi +\tau ){\sigma \pi r}_{0}^{2}} \end{array}$$

Performing comparative studies may warrant the specification of the similarity/difference in the solutions of the classes/compartments at the different equilibriums the underlining model and the new model. For instance at the disease-free equilibrium the solutions derived in the two works [12,13] are similar, but difference is seen at the endemic equilibrium where the parameters for distribution density and transmission range was part of the solutions.

**Fig. 3:**
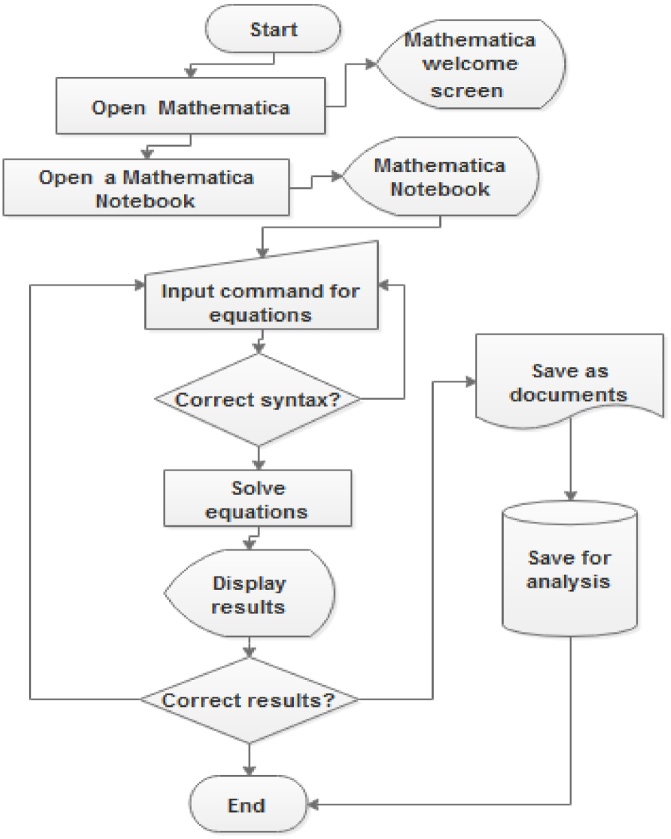
Procedure for using a Solver.

Depending of the complexity of the modeled phenomena, the solutions of the possible equilibriums in wireless sensor networks (or other networks treated like a dynamical system) may prove too daunting and complex to generate by hand. Even though the solutions at the disease free equilibrium seem easy to derive, the solutions at the endemic equilibrium will definitely require the use of solvers such as Maple, Mathematica etc. Fig. 3 depicts the procedure for using a solver in epidemic studies.

### Deriving the basic reproduction number (epidemic threshold)

The focus of researches in epidemiological studies has been to ascertain the tendency/threshold at which an infectious disease may invade/overwhelm a certain population. The basic reproduction number ($R_{O}$) is a measure of the potential for disease spread in a population, and "it represents the average number of secondary cases generated by an infected individual if introduced into a susceptible population with no immunity to the disease in the absence of interventions to control the infection" [27]. Popular methods for finding the $R_{O}$ include; the next generation operator technique described by Diekmann *et al*. [28] and the inverse of the susceptible class at the endemic equilibrium used by Mishra & Pandey [16]. The hypothetical study used employed the latter instead of the former; therefore the Reproduction number is;

(4) $$R_{O}=\frac{\beta \theta {\sigma \pi r}_{0}^{2}}{\left(\theta +\tau )(\nu +\tau +\omega \right)}$$

Here $R_{O}$ is a mathematical expression which involves infection capacity, rate at which exposed nodes become infectious, the effective contact rate (communication range, density) for transfer of infection, rate of recovery, death rate as a result of hardware/software failure and worm infection. The condition for the existence of the endemic equilibrium goes thus: At Ro < 1, the infection dies out and WSN is free of the malicious code, but at Ro ≥1 the worm infection spreads and an epidemic occurs in the network.

In case of comparative studies using epidemic models, the analyst can (aside stating the new reproduction number), also state the reasons behind any obvious similarities/differences when compared with the model that motivated the current study. For instance, since the epidemic threshold of the case study have been stated, it is clear that it involves parameters for distribution density and transmission range which is absent in the epidemic threshold of [12]. The epidemic threshold of [12] is;

(5) $$R_{O}=\frac{\alpha \beta }{\left(\mu +\alpha \right)\left(\mu +\varepsilon +\gamma \right)}$$

In addition, the two reproduction numbers were derived using different approaches yet they gave similar result excepting the newly added parameters for density and range. Specifically, while (5) was derived using the next generation matrix method, (4) was derived by finding the inverse of the susceptible class at the endemic equilibrium. This also shows to a large extent that the reproduction number can be validated by using one approach and checking its accuracy using the other.

### Stability analyses of the equilibrium states

To check for the stability of the equilibrium states most researchers use the Jacobian stability approach to prove the stability, even though they do so for only the disease-free equilibrium state. The Jacobian approach involves finding the Jacobian matrix of the disease free equilibrium state. The disease free equilibrium is locally asymptotically stable if all the eigenvalues of the matrix have negative real parts and unstable if any eigenvalue has a positive real part [29] or the “characteristic equation of the jacobian matrix” derived from the system of equations has negative roots [30]. One common approach in studying the global asymptotic stability of the DFE is to construct an appropriate Lyapunov function and follows the form of La Salle's Invariance principle [15].

In the light of hypothetical case study, the worm-free equilibrium is locally asymptotically stable if $R_{O}$ < 1 and unstable if $R_{O}$ > 1. Using $E_{0}^{F}$ – the characteristic equation of system (1) at worm-free equilibrium is;

(6) $$det\left|\begin{array}{ccccc} –\left(\tau +\rho \right)-x & 0 & \frac{-{{\beta \sigma \pi r}_{0}}^{2}\lambda (\xi +\tau )}{\tau (\xi +\tau )} & \varphi & \xi \\ 0 & –\left(\tau +\theta \right)-x & \frac{{{\beta \sigma \pi r}_{0}}^{2}\lambda (\xi +\tau )}{\tau (\xi +\tau )} & 0 & 0\\ 0 & \theta & –\left(\tau +w+\right)-x & 0 & 0\\ 0 & 0 & \nu & –\left(\tau +\varphi \right)-x & 0\\ \rho & 0 & 0 & 0 & –\left(\tau +\xi \right)-x \end{array}\right|=0$$

(7) $$equating\;to;-\left(x+\tau \right)\left(x+\xi +\rho +\tau \right)\left(x+\tau +\varphi \right)\left(\left(x+\theta +\tau \right)\left(x+\nu +\tau +\omega \right)-S_{0}^{*}\theta {\beta \sigma \pi r}_{0}^{2}\right)=0$$

The roots of the characteristic equation all have negative real parts i.e. –τ, -ξ-ρ-τ, -τ-φ, $\frac{1}{2}\left(-\theta -\nu -2\tau -\omega -\sqrt{{\left(-\theta +\nu +\omega \right)}^{2}+4S_{0}^{*}\theta {\beta \sigma \pi r}_{0}^{2}}\right),\;\frac{1}{2}(-\theta -\nu -2\tau -\omega +\sqrt{{\left(-\theta +\nu +\omega \right)}^{2}+4S_{0}^{*}\theta {\beta \sigma \pi r}_{0}^{2}})$; therefore the worm free equilibrium is locally asymptotically stable.

Although A^2^CDSADM allows for comparative epidemic studies using the differential equation model, our primary interest in this study is not mathematical (stability) analyses. This is because the validation (or Model Alignment and Equivalence Testing) of our agent based model requires results of simulation experiments performed with the benchmark analytical model (BAM) only. These results can be obtained without the stability analyses. Essentially, we intend to go beyond these mathematical analyses to the implementation of spatial factors (and other instances of stochasticity/heterogeneity) not possible with the differential equation method. However, other proofs of local and global stability for the SEIR-V model equilibriums have been addressed in Singh et al. [23].

### Model implementation

The system of differential equation would be solved with the Runge-Kutta-Fehlberg order 4 and 5 method – a suitable numerical method for initial value (IVP) problems. Note that this numerical method was widely used in above listed works. MatLab ode45 built-in function is used to solve the system of differential questions. The analyst may wish to present the algorithm (i.e. pseudocode) for using the built-in function.

Algorithm for the Analytical SIER-V Model

1 Open an mfile and name it seirv1
2 Input function dy = seirv1 (t,y)
3 Specify the column vector
4 Declare the input data values for N, sigma, r, lambda, beta, tau, omega, theta, nu, phi, rho and xi
5 Input differential equation (dy(1)) for Susceptible sensor nodes
6 Input differential equation (dy(2)) for Exposed sensor nodes
7 Input differential equation (dy(3)) for Infectious sensor nodes
8 Input differential equation (dy(4)) for Recovered sensor nodes
9 Input differential equation (dy(5)) for Vaccinated sensor nodes
10 Open the command window
11 Input the syntax for solving the problem [x,y] = ode45 (@seirv1, tspan, y0)
12 Plot the results

### Sensitivity analysis/numerical experiments

Numerical experiments (NE) involve slightly altering the model inputs and checking the corresponding effect on the model output. The importance of NE cannot be over emphasized as far as models are concerned because it can help determine the relative effects of model parameters on model results. Perturbations of the model using several values for the several rates in the proposed model will result in responses which are interpreted.

**Fig. 4:**
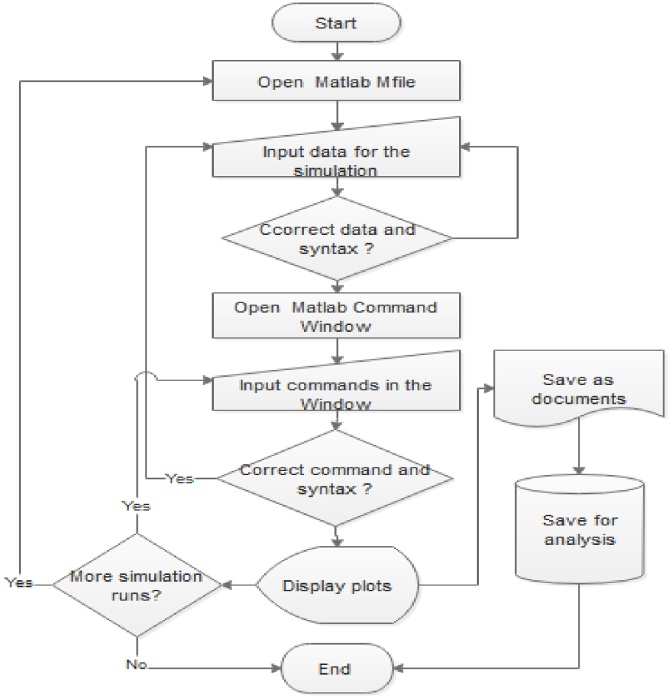
Procedure for using Matlab 7.0.

Here, we performed simulation experiments using the following initial values for the Wireless Sensor network: S = 100; E = 3; I = 1; R = 0; V = 0. Other values used include λ= 0.33, β = 0.1, τ = 0.003; ω = 0.07;θ = 0.25; ν = 0.4; φ= 0.3; ρ= 0.3; ξ = 0.06; adapted from the time history of [12]. The results of varying the parameters of our case study are presented as plots below. Fig. 4 shows the numerical simulation procedure using the above-stated algorithm for the analytical model.

**Fig. 5:**
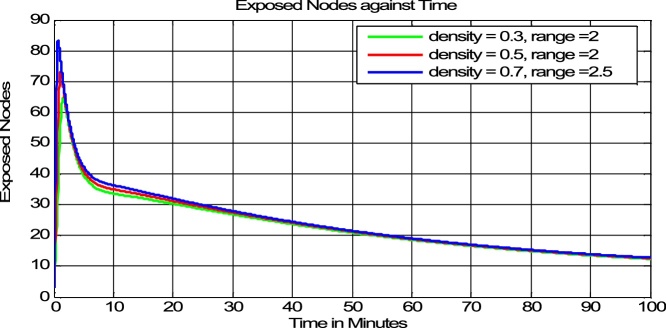
Time History w.r.t. to *σ* and $r_{o}^{2}$.

**Fig. 6:**
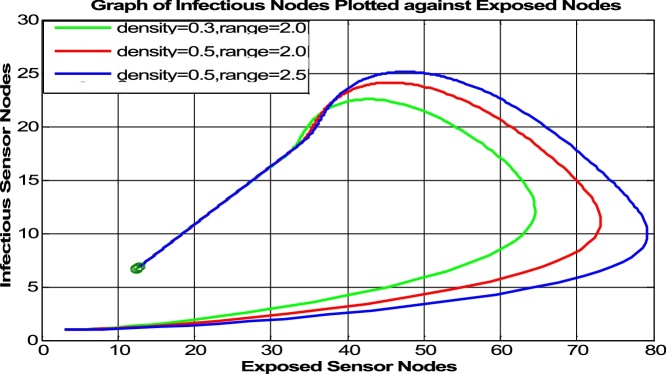
Infectious vs Exposed Class w.r.t. to *σ* and $r_{o}^{2}$.

Fig. 5 depicts the behavior of the Exposed class with respect to time while Fig. 6 represents the dynamical behaviour of Infectious class against Exposed class with respect to varying *σ* and $r_{o}^{2}$. In addition validation can be performed by comparing the results of our hypothetical case study [13] with the results of the model from which it was adapted [12].

**Fig. 7:**
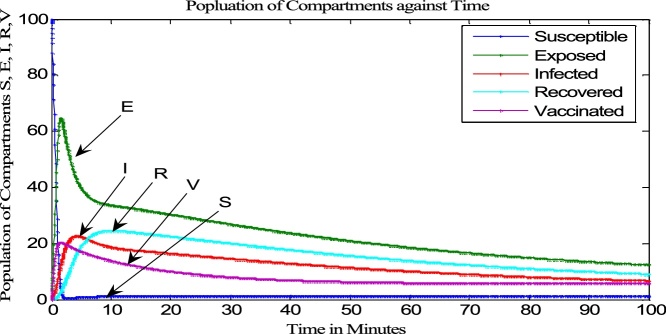
Time History of [13].

**Fig. 8:**
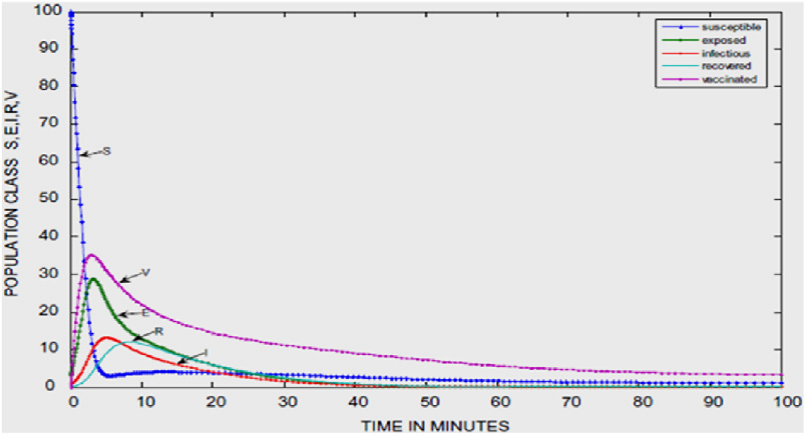
Time History of [12].

**Fig. 9:**
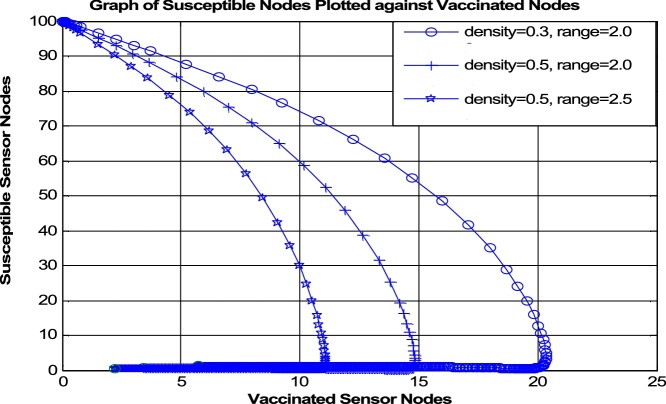
Susceptible vs Vaccinated w.r.t. to *σ* and $r_{o}^{2}$ [13].

**Fig. 10:**
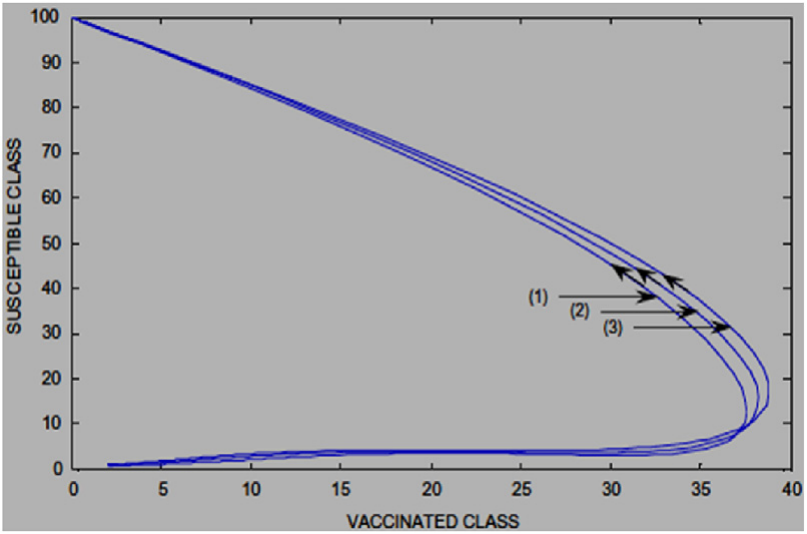
Susceptible vs Vaccinated Class [12].

The impact of including the distribution density and transmission range is evident if the time histories and the graph of the Susceptible class against the Vaccinated class are compared in (Fig. 7 [13] Fig. 8 [12] and Fig. 9 [13] Fig. 10 [12]) respectively.

## Agent-based modeling (ABM) of the SEIR-V model

Agent Oriented Software Engineering (AOSE) – has become one of the active areas of computing research because it involves the creation of software agents or multi-agents bearing in mind concepts such as autonomy, social ability and agent interaction. The steps to the successful application of AOSE have been referred to as *Agent Oriented Analysis and Design* [1]. Wooldridge & Ciancarini [1] asserted that the major roles played by formal methods in software engineering include; the *specification* of systems; for *directly programming systems*; and in the *verification* of systems. These roles encapsulate the core thematic steps of Agent Oriented Analysis and Design or what Jennings & Wooldridge [31] called the Agent Oriented Life Cycle. The concepts of what these roles mean for an ABM or MAS, alongside some addition of steps that ensures the reproducibility of resulting models are presented below.

With the agent based modeling approach, the aim is to *reify* thus *embody* the factors of malware propagation in WSN i.e. building a Sensor Worm Spread Simulator (SWSS) that goes beyond basic mathematical principles to a richer representation of real-world WSN scenarios using the NetLogo agent toolkit. We intend to incrementally modify the developed agent model; and the basis for excellent modification would be justified at first using the already built analytical model (BAM). The following steps constitute the agent modeling approach;

### Additional requirement gathering, analysis and model specification

Firstly, the analyst or modeler (again – for the second time) sources WSN related literature, reviews and understands them. This helps him/her to identify the factors to include in the proposed agent model. Remember, that this agent model must to an extent possess some basic features of the earlier analytical model, if we are to validate it using the analytical model. Secondly, the modeler specifies the requirements of the agent framework and the characteristics to represent. In addition, the prospective agent framework to be developed may be designed as an intentional system that possesses beliefs, goals, actions and the ongoing interaction between agents and their environments; this is formally called the BDI architecture [31].

Under the requirement gathering stage, we firstly identify the basic features of the analytical model, and then other agent factors may follow. Specifically, the development of the agent computational model (or simulator) would require several agents and the WSN environment. On a general note, the model inputs/outputs are determined using the widget of the NetLogo agent toolkit used. Specifically, inputs are sliders, buttons, choosers, switches while outputs are monitors and plots.

A Agents – sensors and malware
B Sensor agents – Susceptible, Exposed, Infectious, Recovered and Vaccinated sensors. Remember that these sensor agents represent first, the state variables of the analytical model.
C Malware agent – Infectious sensor
D Environment – spatially clustered network for the WSN

On the possible beliefs, desires and intentions of the sensor agents, the factors to be implemented include movement, death, ageing. Others include the state variables of the agent model i.e. susceptible, exposed, infectious, recovered or vaccinated. On the scale specification, there is time and size of sensor nodes. The sensor node size is set to 1.5, to enable easy visualization. Time in the model is measured in ticks and is used to measure ageing, life span, death, duration of temporary recovery (immunity) etc; and it is random unlike the equivalent analytical model where time is deterministic. For sensor mobility, the agent model will implement the random walk. Mixing here is heterogeneous, unlike the analytical model that assumes homogenous mixing. As the models would involve a lot of interacting agents (sensors), it became very necessary to represent each class with a color to reduce confusion. Later modifications of the agent model may include other factors gleaned from the requirement gathering and analysis stage.

### Model analysis and design

With the exception of Unified Modeling Language (UML), most of the steps discussed under this section were not originally considered in AOSE. These steps include algorithm design (as pseudocode or flowchart), graphical user interface (GUI) controls and layout design and building the data dictionary. We propose their addition since they are software engineering activities that can aid readability, modifiability and overall reproducibility of the resulting agent model.

A. Unified Modeling Language (UML) analysis

**Fig. 11:**
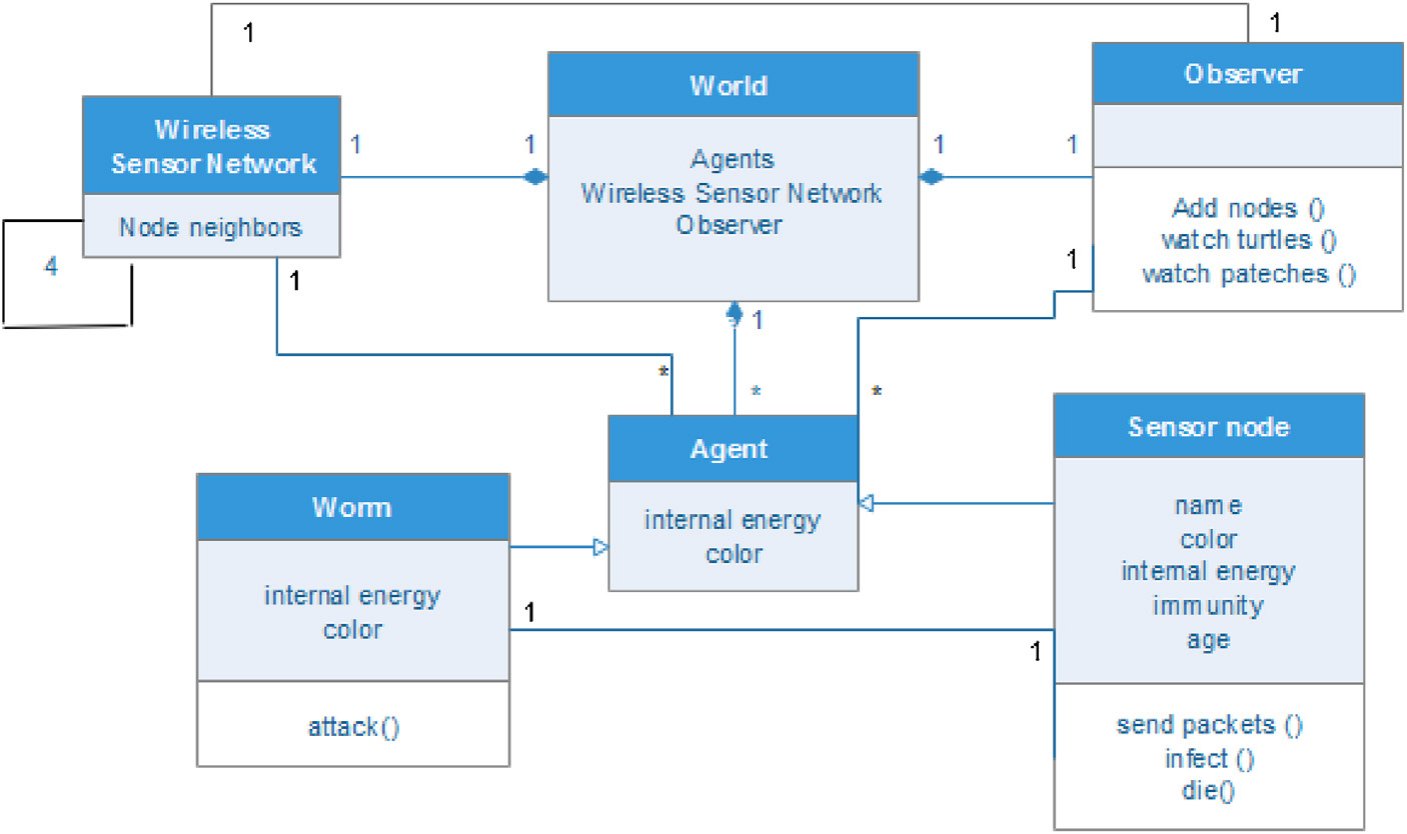
Class UML Diagram.

**Fig. 12:**
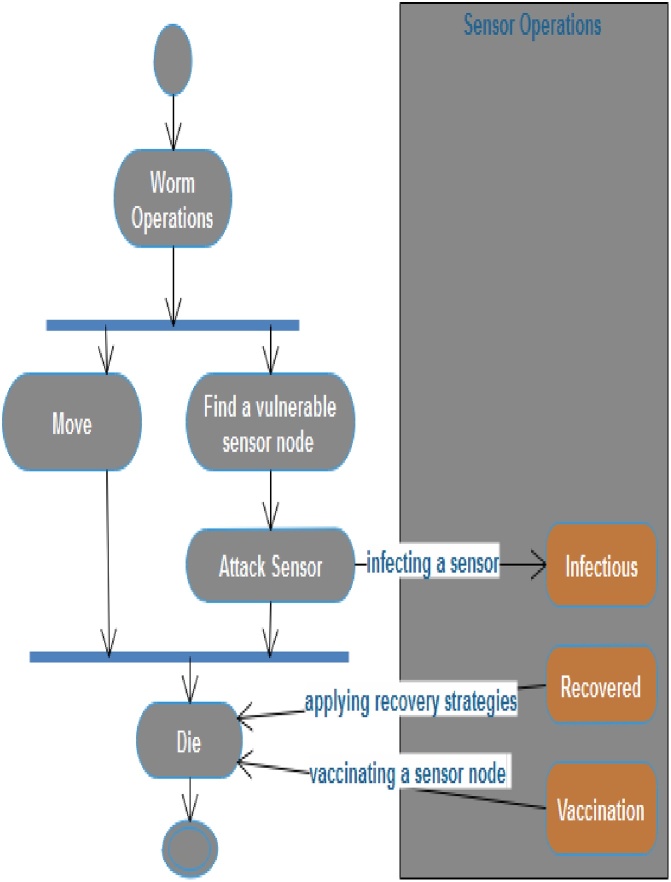
Activity UML Diagram for Worm Operation.

**Fig. 13:**
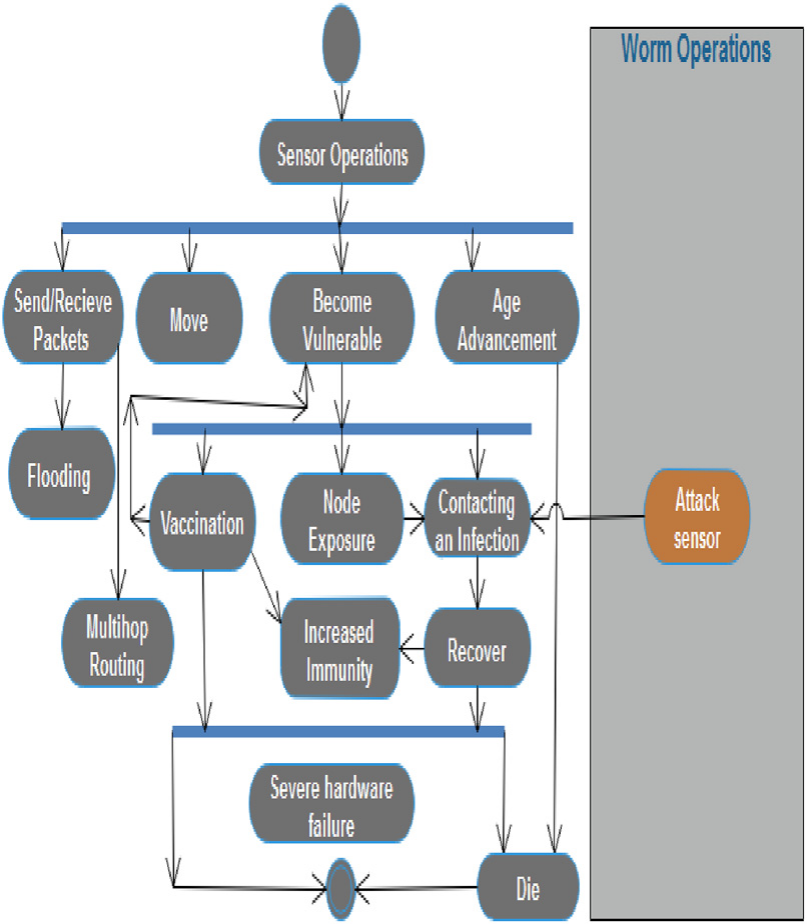
Activity UML Diagram for Sensor Operation.

As Nwokoye et al. [32] puts it, “there is an agreement on the usage of Object Oriented Programming (OOP) concepts (graphically represented using UML diagrams) [[33], [34], [35]], and this is because it presents a natural platform for ABM implementation”. Notwithstanding the modification that necessitated its removal in [36], some authors still insist on the usage of OOP/UML; extending regular OOP objects to what they refer to “*Agent Oriented Programming (AOP)”* [37,38]. Some UML diagrams of WSN are presented as Fig. 11, Fig. 12, Fig. 13. Note that though we only presented class and activity UML diagrams, other diagrams such as the object, sequence, case and class (behavior) UML diagrams can also be used for analysis in ABM.

B. Algorithm

Depending on the needs of the analyst, he/she may want to use pseudocode instead of flowcharts. Under flowcharts, the analyst may have to show both the model and system flowchart. Generally, system flowcharts are a way of displaying how data flows when using the model and how decisions are made to control events therein. Additionally, it can also display work flows and processes in the system. It is evident from the system flowchart below that the user starts the simulation model (or simulator). Then the model parameters of attributes are set in the light of what the user hopes to achieve when the simulator is displayed. Simulating either packet transmission or worm propagation here implies running several underground NetLogo instructions and displaying results as plots. The NetLogo BehaviourSpace tool can be used to rum several simulation experiments which are collated, sorted and saved in a database.

**Fig. 14:**
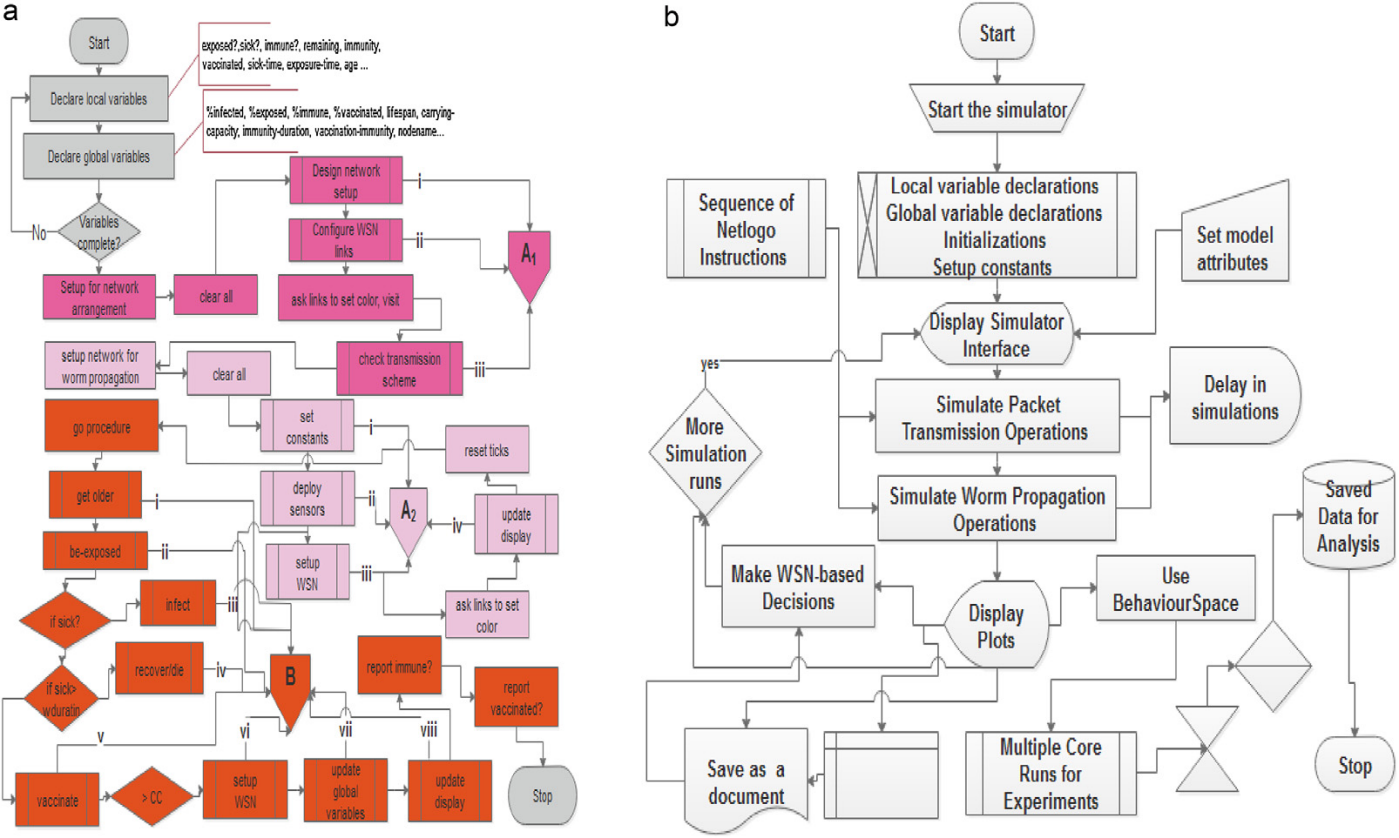
(a) Flowchart for Sensor Worm Spread Simulator. (b) System Flowchart for Model Usage.

Including UML and algorithm (either as pseudocodes or flowcharts) in agent based modeling aids faster and accurate design of the of the GUI controls. Additionally, flowcharts (and activity UML diagrams) are essential, “if one would want to deal with the more procedural instructions flow related parts of the codes” [39]. Furthermore, it aids easy modification of the models at subsequent times; and makes the resulting model reproducible. Fig. 14(a) depicts the flowchart of the proposed agent model while Fig. 14(b) depicts the system flowchart for using NetLogo agent toolkit.

Pseudo-code for the Sensor Worm Spread Simulator

1 Declare local variables for the turtles (sensor nodes and worms)
2 Declare global variables for the turtles (sensor nodes and worms), patches and the observer
3 Set up the procedures
4 Set up the sensor nodes
5 Set up sensor nodes in a wireless sensor network
6 Declare the procedure for a sensor node to be sick and infectious
7 Declare the procedure for a sensor node to be healthy and vulnerable
8 Declare the procedure for a sensor node to recover and become-immune
9 Declare the go procedures for the sensor nodes
  a Run procedure to make the sensors nodes to get older, to recover-or-die if sick?, to infect, if else sick?
  b Run procedure to add more sensors, to update global variable, to update display and to progress tick
10 Update-global-variables
11 Update-display
12 Declare turtle procedure to increase in age
13 To infect ask turtles who are not sick and immune to get sick
14 Run procedure to Recover-or-die
15 Run procedure to Add more nodes if sensors < carrying capacity and random-float 100 < chanceaddnode
16 Show a report on the number of sensors who have recovered and are immune report
17 Startup …setup-constants

C. Design graphical user interface (GUI) controls and layout

**Fig. 15:**
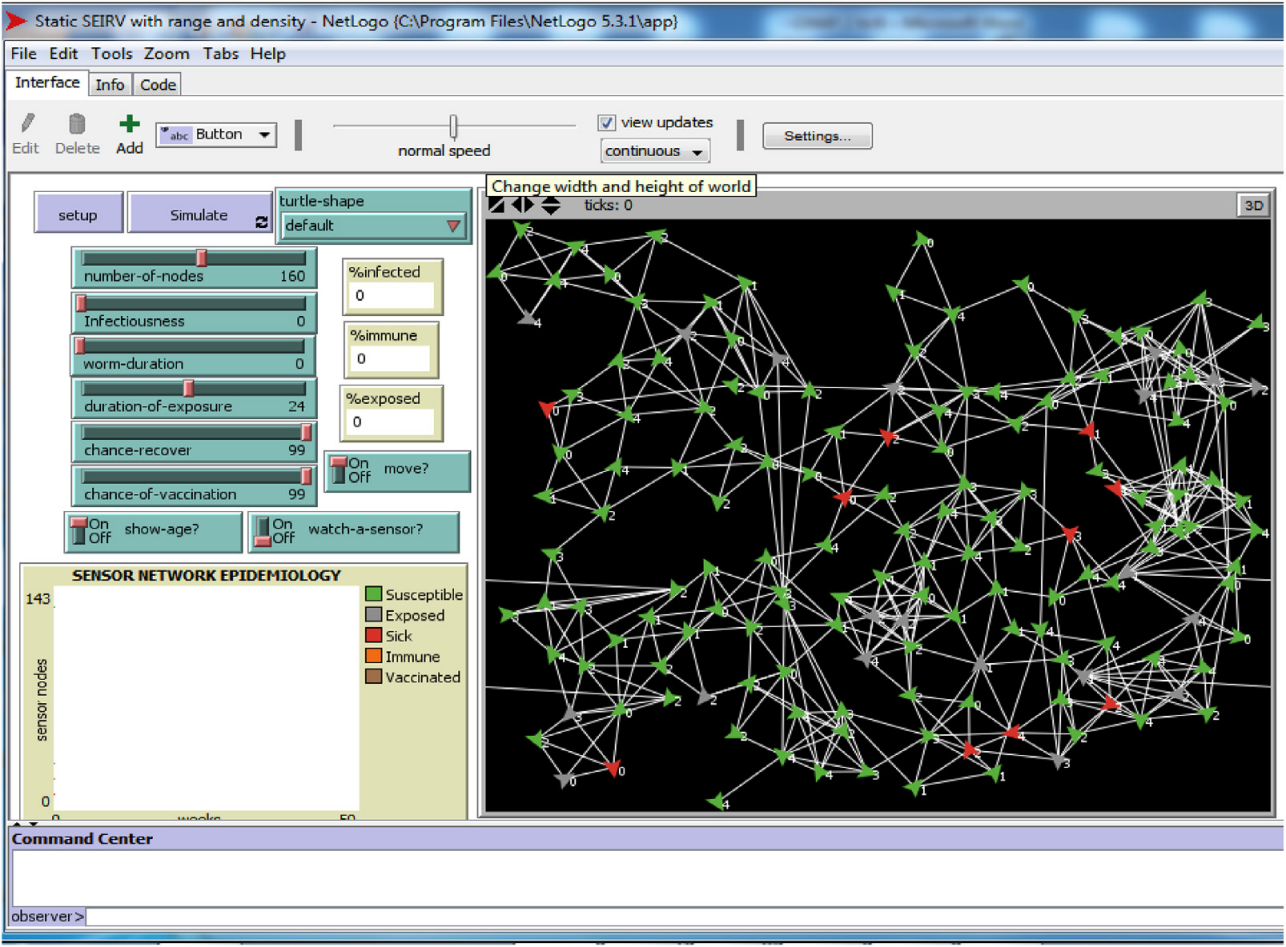
GUI Control Center for the Agent Model.

Here the analyst, designs prototypes of the model’s GUI controls; tinkering on which Netlogo widget suits a particular belief/desire/intention/goal of agent-agent/agent-environment interaction; as well as goals of the end user. The analyst may use a simple rapid prototyping tool like paper prototypes to depict mock up screens. The paper prototypes help represent and evaluate early design ideas. However, one can make the paper prototypes high fidelity by adding little widgets/controls to the NetLogo workspace and coding the underlining capability at once. We also advocate the use of layout tools that aid the user interface design. Fig. 15 shows the GUI layout for the developed agent model.

D. Build the data dictionary

A data dictionary is a collection of descriptions of the data objects or items in a model for the benefit of programmers and others who need to refer to them. The data dictionary would provide information about each attribute of the simulation model. For our models herein, attributes are sliders, buttons, monitors, plots and choosers etc., therefore their names and descriptions would constitute our data dictionary. The descriptions of some model attributes are presented in Table 2 below.

**Table 2:** Data Dictionary for Sensor Worm Spread Simulator.

Attribute Name	Required	Widget	Length	Description
Number-of-nodes	Yes	Slider	300	This controls the number of sensors in the wireless sensor network.
Design Network Setup	Yes	Button	N/A	This allows the deployment and linking of sensor nodes to form a wireless sensor network during packet transmission.
Show-age?	Yes	Switch	On/Off	A switch that allows or disallows the display of a sensor’s age. Note that a sensor dies whenever its age gets bigger than the stipulated life span of a sensor.
%Vaccinated	Yes	Monitor	100	Shows the percentage of the vaccinated sensor nodes from the total sensor population.

### Implementation

At this stage the modeler goes beyond abstract specification to concrete implementation (i.e. a computational system). Executing or animating the specification may involve some level of programming – *agent oriented programming (AOP)*. This term was coined by [40]; and used to describe a specialization of (OOP). He maintained that, “AOP specializes the framework by fixing the state (now called *mental state)* of the modules (now called *agents)* to consist of components such as beliefs (including beliefs about the world, about themselves, and about one another), capabilities, and decisions, each of which enjoys a precisely defined syntax”.

Coding the agent model should be done alongside the design of the graphical user interface (GUI) controls/layout and data dictionary development. At this juncture the modeler writes rules/instructions (according to the syntax of the toolkit) that animate the earlier specifications made.

### Verification and validation

**Fig. 16:**
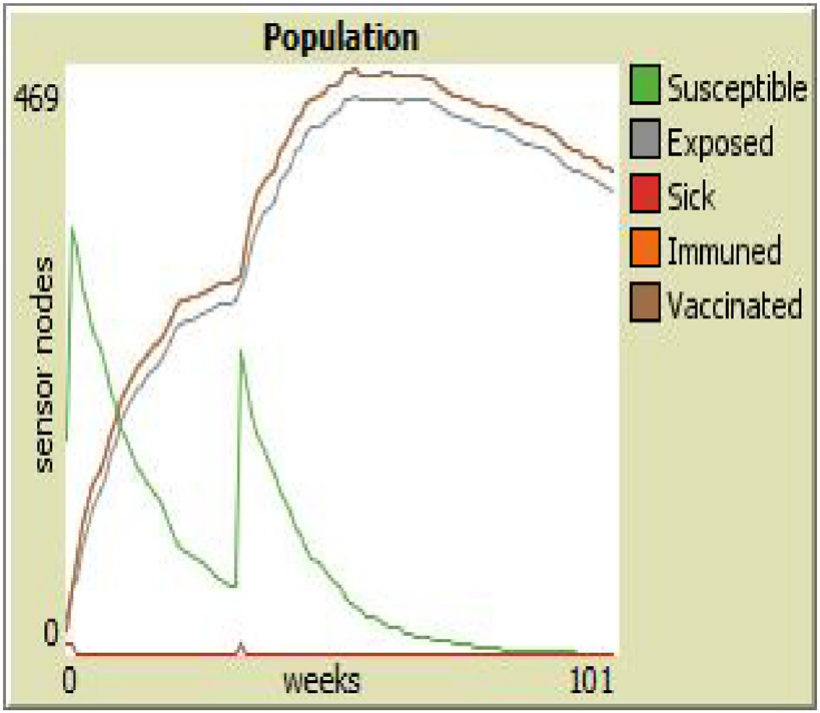
Infectiousness and Worm duration is 0.

**Fig. 17:**
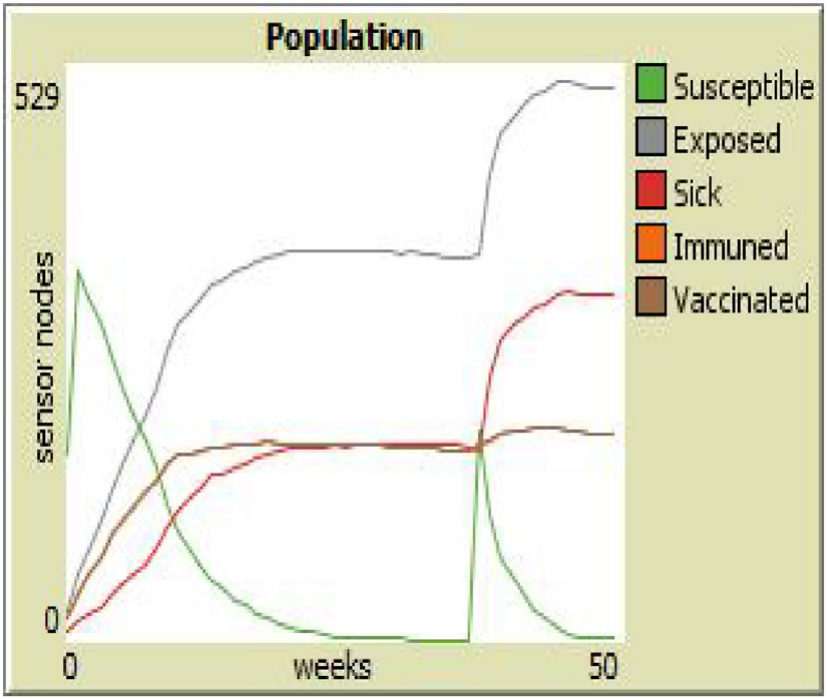
Infectiousness and Worm duration is 99.

There is need to show that this developed system is accurate in the light our original specification. This process is known as *verification*, and it is specifically essential for the development process. Jennings et al, the stage is divided into two broad classes: (a) *axiomatic*; and (b) *semantic* (model checking) and since, axiomatic methods possess some limitations (syntactic proof problems), model checking is preferably used because it is based on the semantics of the specification language. The developed models are tested, and used to make predictions. Fig. 16 shows the result of the agent model when Infectiousness and Worm duration is 0, while Fig. 17 shows the result of the agent model Infectiousness and Worm duration is 99.

### Equilibrium generation

With the help of A^2^CDSADM, visual essences of our analytical equilibrium solutions are generated using the simulator (or agent model). These visual essences/equivalents will not only depict the health status of the sensor nodes (using colors) but show possible spatial orientation of the sensors in the WSN environment.

**Fig. 18:**
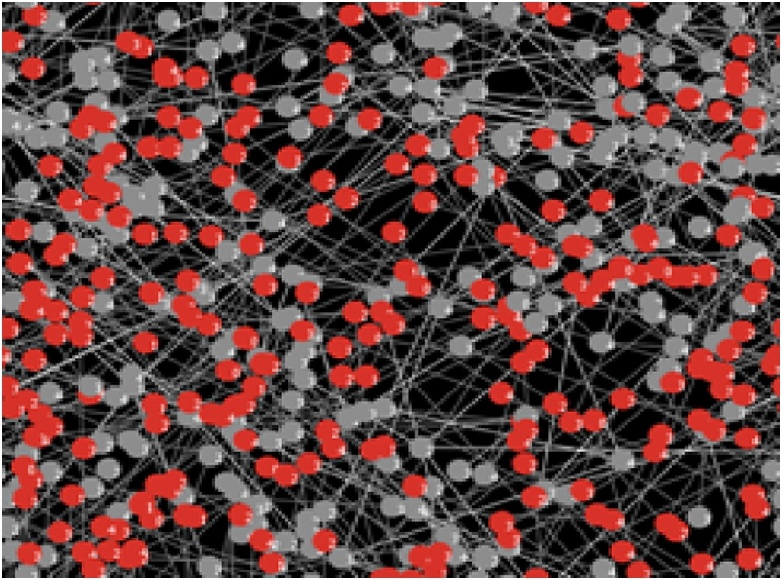
Endemic Equilibrium for SWSS.

**Fig. 19:**
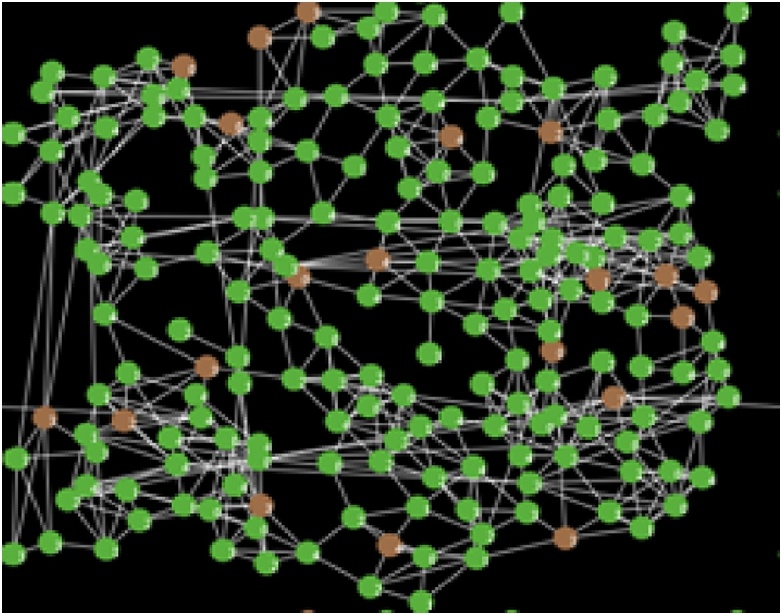
Malware-free Equilibrium for SWSS.

Given the colors of different sensor nodes in Sensor Worm Spread Simulator (SWSS), Fig. 18 shows the endemic equilibrium in the wireless sensor network environment. This is a point in which most sensor nodes carry the infection. The grey nodes (which are the exposed sensor nodes) will soon turn red and be ready to pass the infection to other susceptible nodes if any is deployed. On the other hand, Fig. 19 shows a malware-free equilibrium in the network environment. This is a point in which most sensor nodes do not carry the infection (i.e. most nodes are either susceptible or vaccinated). Fig. 19 is what most organization that use wireless sensor network strive to achieve for the achievement of their meaningful daily activities.

### Model alignment and equivalence testing (MAET)

Model alignment is an evaluation approach, and as [3] puts it, “it involves aligning both computational models or ‘docking’ to match the output of the proposed agent-based models to that of the epidemic models using available information about the malicious-code spread characteristics”. The essentiality of model alignment and equivalent testing is captured in two words, “critical experiment and subsumption” [41]. If two models (with distinctively different modeling approaches) attest to representing the same phenomena, then it is fundamental to know whether they can/cannot generate same results. This is the rationale for performing “critical experiments and for tests of whether one model can subsume another” [41]. Successful MAET process encourages justified “subsumption” i.e. rejecting the earlier (analytical) model in favor of the later (agent) model that (perhaps) has a richer representation or saying the later model is a significantly exceptional expression of a traditional one.

From the graphical abstract, model alignment and equivalent testing may seem like the final stage of the A^2^CDSADM. However, if the analyst/modeler wants to incrementally modify the agent model to a richer and fuller realization of WSN epidemiology, then it is not. Simulation results are compared, to gauge the correctness of the agent model (or simulator) by validating it against the analytical SEIR-V model. Although this may not be entirely sufficient, but achieving (result equivalence) reflects some sort of convincing plausibility for the simulator and establishes confidence that may lead to its further modification and validation.

**Fig. 20:**
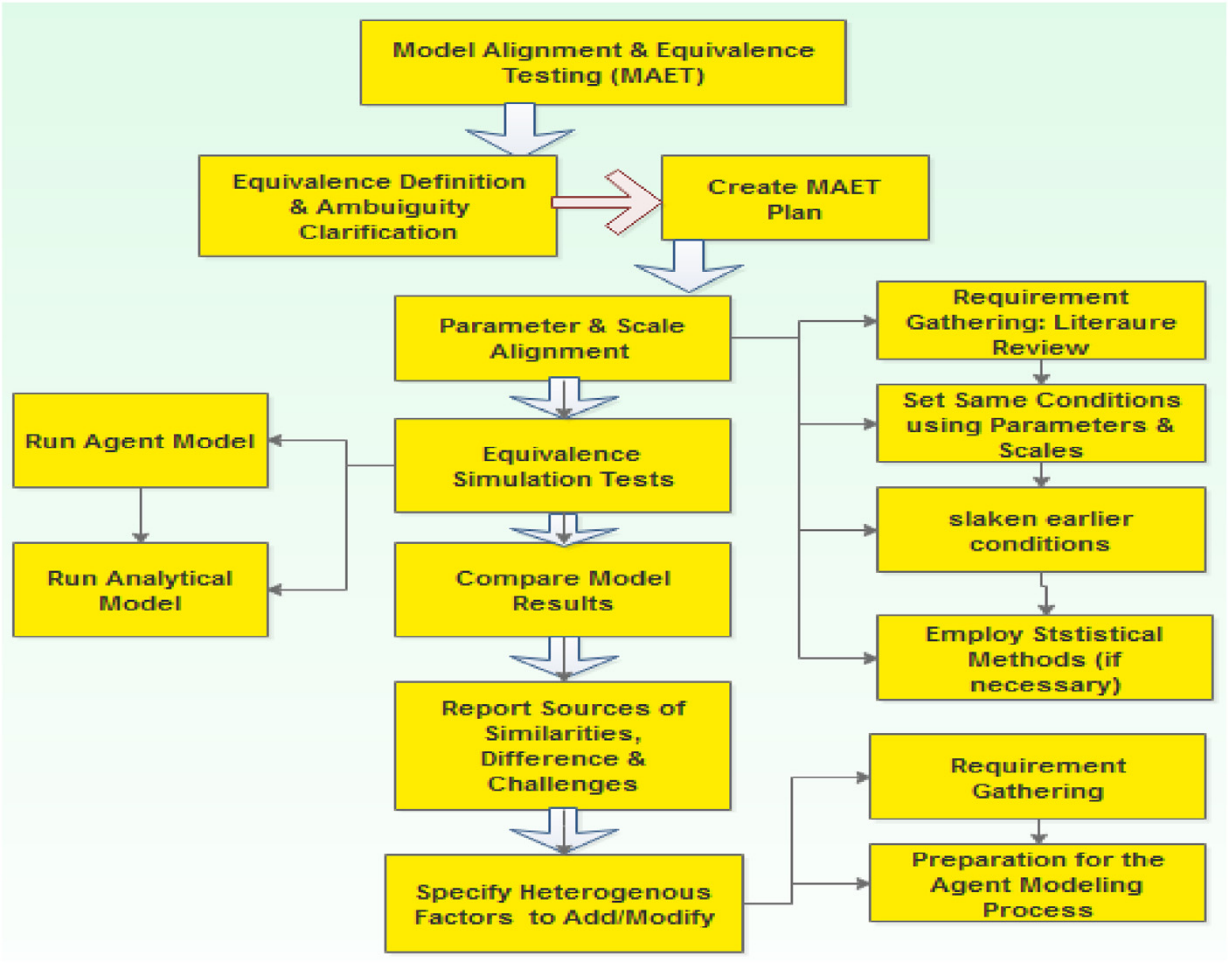
MAET process for A^2^CDSADM.

Fig. 20 describes a formalized MAET process for A^2^CDSADM that aids easier modification of the agent model. The MDET process in Fig. 20 consists of seven major steps; 1. *Equivalence Definition and Ambiguity Clarification*. 2. *Create a MAET plan*. 3. *Parameter and Scale Alignment*: this stage may include literature review, setting same conditions in accordance to review details, slackening the earlier conditions and statistical activities for equivalence determination. 4. *Equivalence Simulation Tests*: this is the actual running of the two models. 5. *Compare Model Results*. 6. *Report Reasons/Sources of Similarities, Differences, Challenges*. 7. Specify more Heterogeneous Factors to Add/Modify: this stage would involve requirement gathering in preparation for going through the modeling process again.

**Fig. 21:**
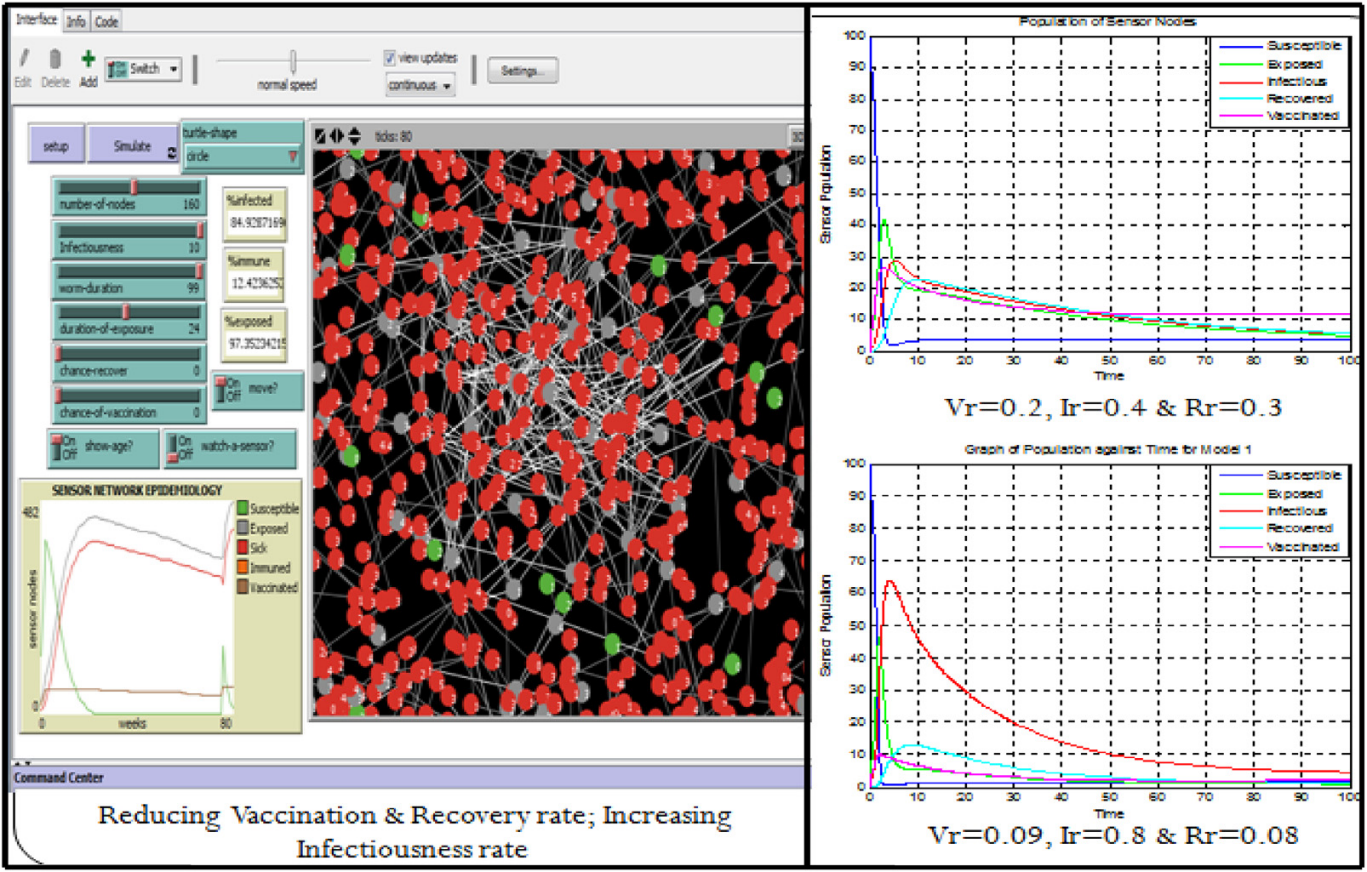
Model Alignment using the Results of Both the Simulator and the Analytical Model.

Specifically, we simulated the effect of reducing the vaccination and recovery rate; and increasing the infectiousness rate. On the right hand side (of Fig. 21) is the result of using the Sensor Worm Spread Simulator (the agent-based model); while on the left hand side (of Fig. 21) is the result of performing the above operation using the analytical SEIR–V model. On the simulator, we set the “*Infectiousness*” and “*worm-duration*” sliders to 99 and setting the “*chance-recover*” and the “*chance-vaccination*” sliders to 0; correspondingly reduced the number of susceptible and increased the number of infectious (sick) sensor nodes in the network environment. Specifically, on the analytical model the vaccination rate was reduced from 0.2 to 0.09; the recovery rate was reduced from 0.3 to 0.08 and infectiousness rate was increased from 0.4 to 0.8.

To some extent the (curves) results from both approaches are approximately the same, at least in the increase in the infectious nodes and the reduction of the susceptible nodes. On the plot of the simulator (at the right hand side of Fig. 21), the susceptible sensor nodes was seen to rise again at the 80^th^ week. This is due to the deployment of a new set of sensor nodes when the number of sensors goes below the carrying capacity of the WSN environment. Subtle differences in the results may be related to stochasticity and heterogeneous mixing of agents in the WSN.

## Additional information

**Fig. 22:**
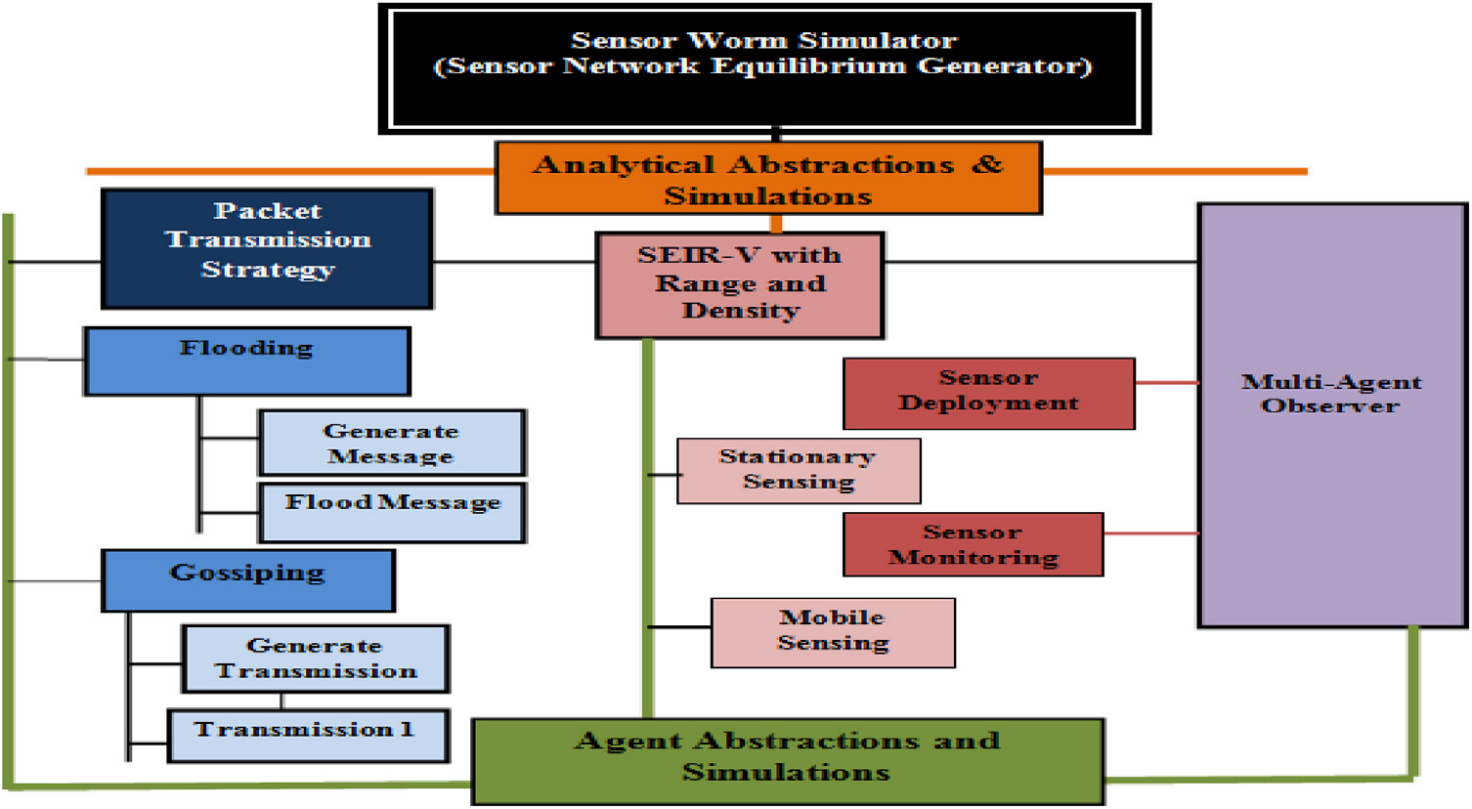
High Level Model for WSN.

The analyst/modeler may decides to approach the A^2^CDSADM using the vertical division view; this would entail performing the activities in each of these stages, namely; *Requirement Gathering and Analysis*; 2. *Model Specification and Formulation*; 3. *Model Analyses and Design* and 4. *Implementation: Simulation, Verification and Validation.* However, in between the two modeling approaches is the high level model (Fig. 22). Alongside performing requirement gathering and analysis, the analyst should create the high level model. This is a unified conceptual model that describes the features to be represented/characterized using both approaches of the A^2^CDSADM. In view of our hypothetical case studies, Fig. 22 represents the high level model for WSN. Note that the high level model should be updated as modifications are made to the resulting agent model as per the addition of more features.

**Fig. 23:**
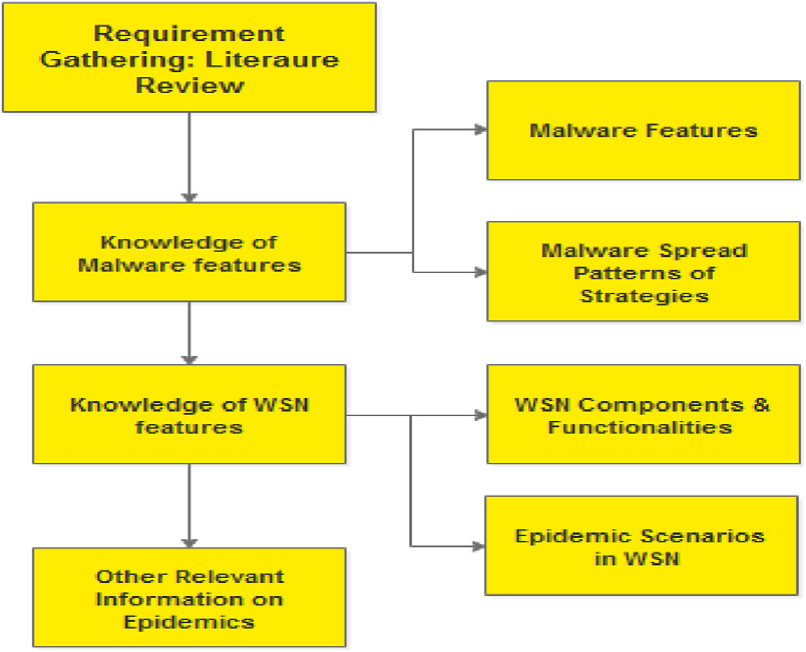
Requirement Gathering in a WSN Context.

Requirement gathering and analysis is a process of sourcing, reviewing and understanding pertinent literature of a real world phenomenon in order to identify the factors/characteristics to be included during model specification and formulation. To accurately perform epidemic studies in WSN, the requirement gathering and analysis stage must elicit firstly the malware characteristics and its spread patterns/strategies; and secondly the WSN characteristics/components as well as other relevant information on epidemics (as shown in Fig. 23). Then in the light of the analyst’s mathematical ability, these characteristics are formulated using equations. The analyst should thoroughly tinker on the generated information so as to ensure that only the relevant features are represented; this is the rationale for including “analysis” in the requirement gathering process. With proper requirement analysis and gathering, the method can be used to model epidemics in other networks.

**Fig. 24:**
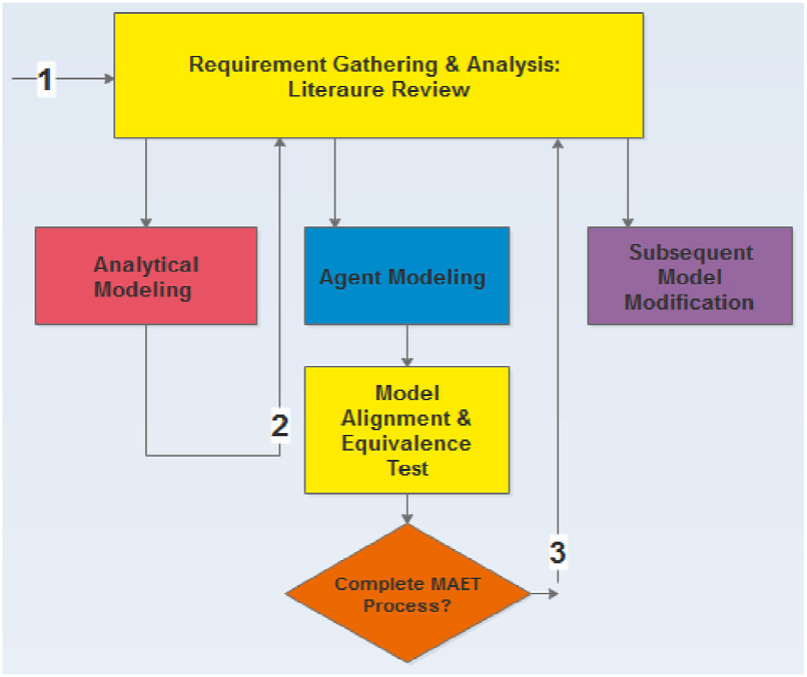
Iterative Process of Requirement Gathering.

Since, we intend to achieve the entire accurate representation of WSN features and scenarios, requirement gathering becomes an iterative process that begins before model formulation and may not end, if one intends to incrementally modify the agent model. The process ends at the point the analyst/modeler decides that the resulting model can be used for accurate decision making on issues related to the modeled phenomena. Fig. 24 shows the iterative process of requirement gathering and analysis. Arrows 1, 2 and 3 pointing upwards depicts the times when the analyst/modeler performs the activities depicted in Fig. 23; while the arrows pointing downward signifies the series of activities that constitute the modeling approaches within A^2^CDSADM).

**Fig. 25:**
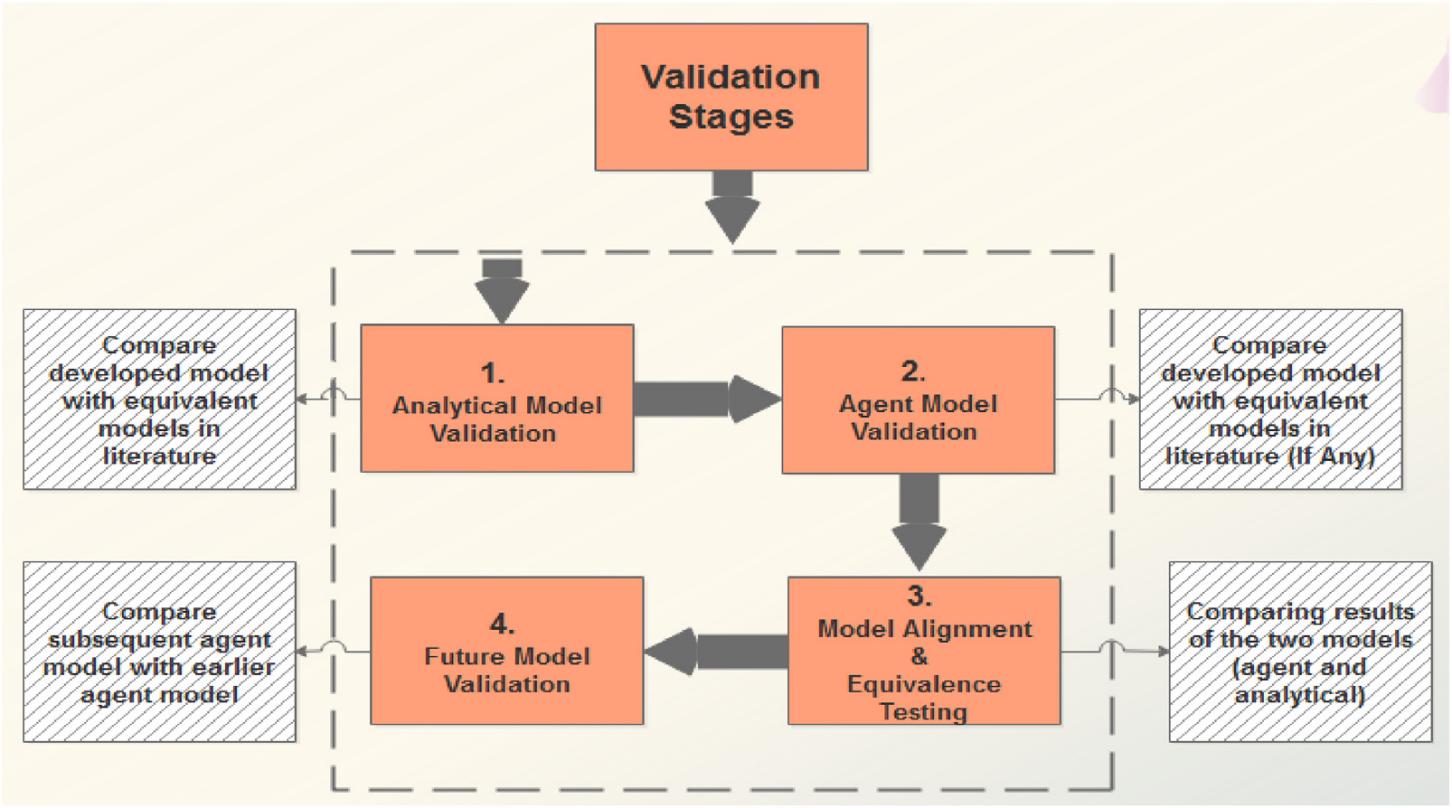
Validation Stages for A^2^CDSADM.

Model validation in the A^2^CDSADM, is a continuous task that aims at generating an accurate model. The points of validation are labeled with numbers from 1 to 4 in Fig. 25. Assuming the analyst does not want to modify the agent model further, the validation ends at stage three; otherwise the 4^th^ stage validation stage is performed. The validation stages include: 1. Analytical Model Validation: compare developed analytical model with equivalent analytical models in literature; 2. Agent Model Validation: compare developed agent model with equivalent agent models in literature (if any); 3. Model Alignment & Equivalence Testing (MAET): compare results of the two models (agent and analytical) and 4. Future Model Validation: compare subsequent agent model with earlier agent model. Our validation ended at the stage 3, this is due to the absence of an equivalent agent model in literature. Note also that stage 2 (i.e. Agent Model Validation) was omitted. After MAET, the future modification of the agent model necessitates the 4^th^ stage of validation.

Beyond the stochasticity that may be achieved by the agent model specifications, epidemic studies can be extended to involve the heterogeneity observable in a real world WSN. The heterogeneity can be in different sizes for the sensors, where a larger sensor node signifies more battery power and smaller sensor node signifies less battery power. The sensors closer to the base stations can have more battery power/energy as they are the cluster heads. Additionally, sensor networks can also be heterogeneous in terms of computational capability of the sensor nodes, bandwidth capability of the links and the initial energy of the sensor nodes. The sensor nodes can be made to send and receive packets

Depending on the intended objectives, an analyst can go beyond the specifications made herein to include factors of cognition, self-organization and cooperation in the attributes of the developed agent based model. More so, adapting the OOD protocol to the A^2^CDSADM by adding parameters for learning, adaptation and prediction (if necessary). Additionally, the analyst can also specify agent roles or protocols of interactions between the agents or between the agent and the environment. Note that in comparative epidemic studies using analytical models, the output of any stage of vertically approaching A^2^CDSADM are compared to its equivalent(s) in literature.

## Malware propagation using individual-based models: state of the art

In order to understand malware spread in telecommunication/technological networks equations-based models have been used to characterize the dynamics of interaction. These models in the light of their characteristics are basically seen in literature as deterministic/stochastic, continuous/discrete and global/individual models etc. The bulk of available models are deterministic/stochastic and continuous/discrete and they pursue the global perspective of evolution wherein the overall population dynamics are investigated. From recent studies [11,42], it has become very clear that malware propagation models based on differential equations are plagued with several shortcomings and drawbacks, irrespective of its successes and popularity in both biological and network epidemiology. The noted drawbacks include homogenous mixing and distribution, inability to represent individual dynamical behavior and the inability to account for local infections between nodes in a network [11]. In other words, “models based on differential equations fail to capture the local characteristics of spreading processes, nor do they include interaction behaviors among individuals” [43].

Researchers suggested the use of Individual based models (IBMs) in order to salvage the above shortcomings. IBMs attempts to highlight the real-world autonomy of interacting individuals/hosts. Studies involving IBMs simulate local interactions between cells/agents in discrete time and space so as to produce emergent outcomes. Examples of IBM are cellular automata (CA) and agent based models (ABM) [42,43]. Few of these models exist for malware propagation, perhaps due to the complexity of representing individual level mechanisms of a particular phenomenon. Though both CA and ABMs model individual level representations, their emphasis is a little different. While CA focuses on emergent outcomes of local interactions, in ABM agents take actions based on their locally coded individual behaviours and that of the environment [44]. Perhaps, more advantage lies with ABM obviously due to the complex representation of explicit behavioral processes.

CA is a discrete, deterministic mathematical model, where space, time, and the state of the system are distinct [43]. It was used to model malware propagation in wireless sensor networks [45] and on smart phones/mobile devices [43,[46], [47], [48], [49]]. Using ABM, Bose & Shin [50] built a framework for malware spread in a heterogeneous environment while Hosseini et al. [51] modeled the outbreak of malware based on the rumor diffusion process.

## Conclusion

Motivated by the sameness in connectivity realities between biological viruses and malware equivalents, we sought to study the spread of malicious codes in close-to-real world WSN environment. Due to the limitation of the traditional analytical model which becomes less tractable with the addition of spatial features in WSN epidemiology, we complimentarily designed equivalent agent model that at first provided visualization and spatial orientation for the deployed sensor nodes. Furthermore, it reified other factors such as carrying capacity, sensor mobility, packet transmission etc.

These models served as hypothetical cases for merging the benefits of two distinctively different modeling approaches that resulted to a hybrid method, coined as the *Analytically-Agent Cyber Dynamical Systems Analysis and Design Method (A^2^CDSADM).* This method brings more to the table; it combines the modeling of cyber dynamical (defense) systems (i.e. networks) with Agent Oriented Analysis and Design (AOAD) – an extension of the Object Oriented Analysis and Design (OOAD)/Object Oriented Software Engineering. Its coding/implementation required the application of new perspective in software engineering called the Agent Oriented Programming (AOP) – an extension of the Object Oriented Programming (OOP).

The methods that constitute A^2^CDSADM were extended to cover the additional features for generating the (analytical) equilibriums of the modelled system (i.e. wireless sensor network), for ensuring model specification accuracy by performing model alignment and equivalence tests, for creating a high level conceptual model containing the envisaged WSN features to be represented, for representing more complex factors of a real world WSN scenario; and to thereafter compare the result of two models.

This method helps achieve the complementary and generative contribution of ABM to analytical modeling, provides a formalized method for performing comparative epidemic studies and aids incremental modification and reproducibility of the agent model in order to achieve a realistically richer representation of the modeled phenomena. It also alleviates the lack of field data/lack of real geographical locations of the occurrence of particular cases by the creation of a benchmark model used for validation.
